# Supplementary material for: Real and Deepfake Face Recognition: An EEG Study on Cognitive and Emotive Implications
Source: Brain Sci. 2023 Aug 23;13(9):1233. doi: 10.3390/brainsci13091233 (PMC10526392; doi:10.3390/brainsci13091233)
Supplement: Supplementary file 1 [file brainsci-13-01233-s001.zip › brainsci-2536962-supplementary.pdf]

Supplementary Materials of “Real and Deepfake Face Recognition: an EEG Study on Cognitive and Emotive Implications”

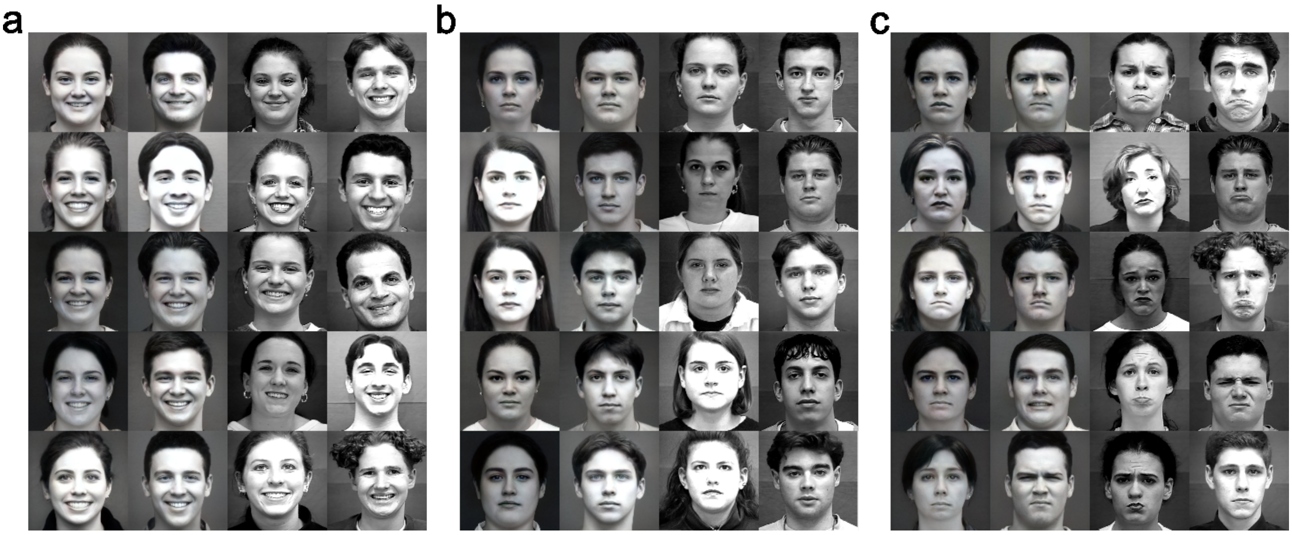

Figure S 1 – All 60 images of human faces used in the proposed visual stimuli task. ***a*** *Happy set,* ***b*** *Neutral set,* ***c*** *Sad set. For each set, the leftmost column report real faces of woman, then faces of real men, AI-generated faces of woman and, finally, AI-generated faces of men.*

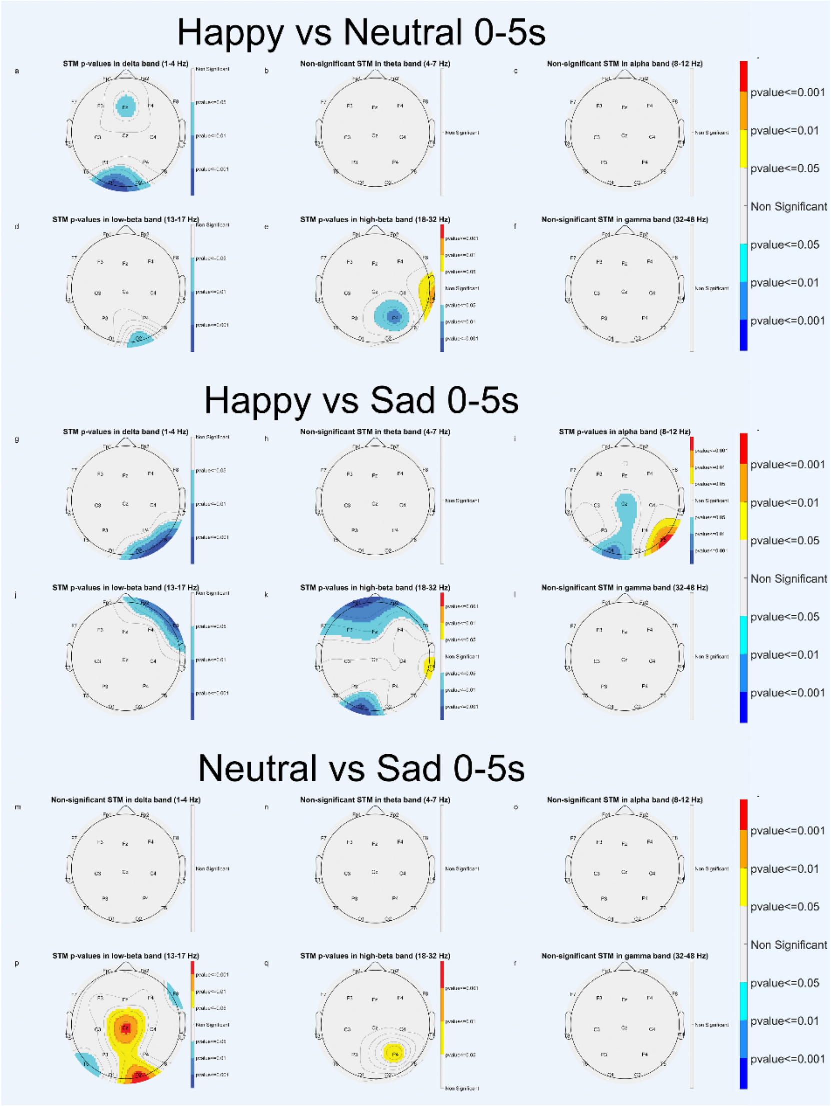

*Figure S 2 – STMs of significant statistical activation of brain areas in Emotional comparisons (0-5 s). "Happy vs. Neutral" comparison (**a,b,c,d,e,f**); **a** delta band, **b** theta band, **c** alpha band, **d** low-beta band, **e** high-beta band, **f** gamma band. "Happy vs. Sad" comparison (**g,h,i,j,k,l**); **g** delta band, **h** theta band, **i** alpha band, **j** low-beta band, **k** high-beta band, **l** gamma band. "Neutral vs. Sad" comparison (**m,n,o,p,q,r**); **m** delta band, **n** theta band, **o** alpha band, **p** low-beta band, **q** high-beta band, **r** gamma band.*

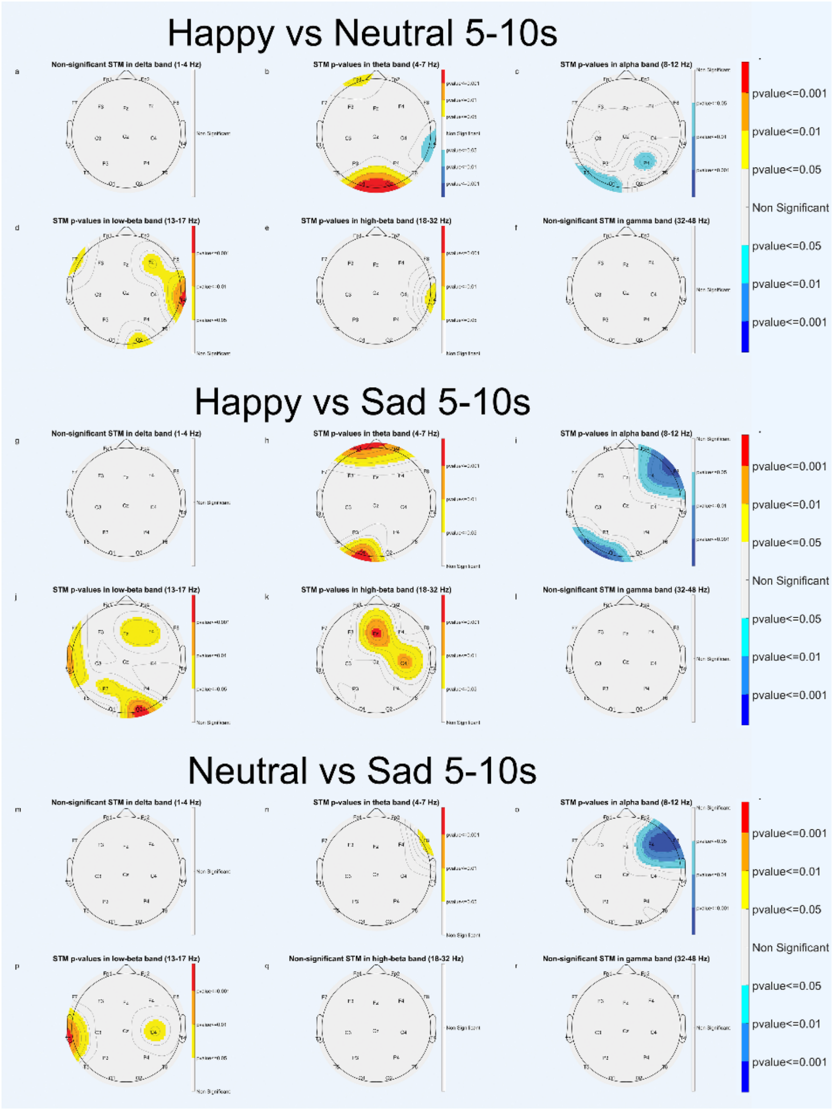

*Figure S 3 – STMs of significant statistical activation of brain areas in Emotional comparisons (5-10 s). "Happy vs. Neutral" comparison (**a,b,c,d,e,f**); **a** delta band, **b** theta band, **c** alpha band, **d** low-beta band, **e** high-beta band, **f** gamma band. "Happy vs. Sad" comparison (**g,h,i,j,k,l**); **g** delta band, **h** theta band, **i** alpha band, **j** low-beta band, **k** high-beta band, **l** gamma band. "Neutral vs. Sad" comparison (**m,n,o,p,q,r**); **m** delta band, **n** theta band, **o** alpha band, **p** low-beta band, **q** high-beta band, **r** gamma band.*
